# Outcomes of Infants With Severe Refractory Food Protein-induced Allergic Proctocolitis Treated With Mesalamine

**DOI:** 10.1097/PG9.0000000000000024

**Published:** 2020-12-17

**Authors:** Maria Belen Rojas Gallegos, Karen D. Crissinger

**Affiliations:** From the Department of Pediatrics, University of South Alabama, Mobile, AL.

**Keywords:** food protein-induced allergic proctocolitis, mesalamine, gastroesophageal reflux disease, elemental formulas

## Abstract

This retrospective chart review evaluates the outcomes of mesalamine treatment in infants with severe food protein-induced allergic proctocolitis (FPIAP) and persistent clinical symptoms despite the use of elemental formulas. Patients received mesalamine in a 40–60 mg/kg/d dose for an average of 100 days. This group showed significantly higher rates of improvement in the most common symptoms of FPIAP compared with the control group. In addition, the mesalamine group was less likely to need pharmacological treatment for gastroesophageal reflux disease and more likely to successfully transition to whole milk or soy milk after 1 year of age. In conclusion, using mesalamine can be a useful addition to the treatment of severe refractory cases of FPIAP.

**What Is Known?**

- FPIAP causes inflammation of the distal colon in response to food proteins, most commonly cow’s milk and/or soy protein.
- Most patients improve following the withdrawal of the suspected food antigen.
- There is a lack of guidelines for infants with a persistent fecal occult blood test and/or clinical symptoms despite the use of elemental formulas.

**What Is New?**

- A short course of mesalamine helped improve symptoms in severe refractory FPIAP cases.
- Using mesalamine reduced the requirement for medically treating GERD.
- Patients treated with mesalamine were less likely to report problems after transitioning to soy or cow milk.

**F**ood protein-induced allergic proctocolitis (FPIAP) has become an increasingly more frequent health care concern. This disorder is a benign transient condition that can present as early as the first week of life but is usually diagnosed within the first few months after birth (1). Usual symptoms include rectal bleeding and stool changes, often described as loose, frothy, and mucousy. More severe cases present with irritability, feeding intolerance, vomiting, and weight loss or failure to thrive (2–4).

Flexible sigmoidoscopic evaluation with a biopsy is a useful tool for evaluating patients presenting with an unclear presentation, severe symptoms, and failure to improve with standard management. Gross findings may include mild colitis with patchy erythema and edematous mucosa with loss of vascularity. Lymphonodular hyperplasia on the distal colon is also frequently observed. Biopsies typically reveal high numbers of eosinophils in the lamina propria and muscularis mucosa (5–7).

There is a lack of evidence-based guidelines for infants with severe FPIAP and persistent clinical symptoms despite the use of elemental formulas.

Mesalamine (5-aminossalicylic acid derivative) is a locally active, anti-inflammatory agent used to manage colitis. There is little information about its use and outcomes in infants with FPIAP.

This study aims to evaluate the outcomes of a short course of mesalamine treatment in infants with severe refractory symptoms of FPIAP.

## METHODS

Data were obtained from all the patients diagnosed with FPIAP from January 2009 to December 2012 for a total of 580 patients. From this pool of patients, we identified those who met the following criteria: less than 12 months of age with no other comorbidities, treated with elemental formulas for more than 2 weeks, and having persistent severe symptoms.

The severity of the symptoms was obtained from parental reports. To be identified as severe, they needed to generate significant distress on patients/parents and routine disruption.

These patients were classified as severe cases and totaled 65. All severe cases underwent an esophagogastroduodenoscopy (EGD) and flexible sigmoidoscopy (FS).

From the group identified as severe, we compared the infants treated with mesalamine (total of 44) to severe cases not treated with mesalamine (total of 21). Patients received medication in the form of capsules opened for microbeads.

We performed a retrospective chart review with a central tendency analysis of numeric data. Statistical significance was obtained using Fisher exact test, and a two-tailed *P* value with significance at *P* <0.05.

## RESULTS

Medical record review from January 2009 to December 2012 showed that 65 of 580 (11.20%) patients met our inclusion criteria. These infants ranged from 15 days to 8 months of age (mean 2.98 ± 1.88 months). Most infants were males (38/65; 58.46%) and Caucasian (51/65; 78.46%). The mean birth weight was 3.15 ± 0.66 kg. There were 48/65 (73.84%) term infants and 17/65 (26.15%) preterm infants.

The average age at the initial evaluation was 2.98 ± 1.88 months old. Most parents reported the onset of symptoms at 18.63 ± 23.19 days of age and had an average of four formula changes before introducing an elemental formula. Patients were on elemental formulas for an average of 1.5 ± 2 months. The most common symptoms reported are described in Table 1.

**TABLE 1.** Symptoms reported in patients assigned as severe FPIAP

Symptoms	No. of patients (%) (N = 65)
Skin problems*	23/65 (35.38%)
Fussiness	60/65 (92.31%)
Choking/Gagging	48/65 (73.85 %)
Decreased appetite/refusal to eat	31/65 (47.69%)
Spitting up/vomiting	63/65 (96.92%)
Back arching	32/65 (49.23%)
Respiratory problems†	32/65 (49.23%)
Changes on stool consistency‡	57/65 (87.69%)
Visible blood or mucous in stool	43/65 (66.15%)
Gassiness	36/65 (55.38%)
Hiccups	41/65 (63.08%)
Sleeping difficulties	12/65 (18.46%)

*Symptoms of atopic dermatitis and xerosis cutis

†Symptoms of persistent cough and congestion not related to another pathology.

‡Watery or hard stools.

On FS, 35/65 (53.84%) patients had evidence of lymphonodular hyperplasia in the rectosigmoid colon, 26/65 (40%) patients were normal, and 4/65 (6%) had patchy colitis. Biopsies from FS showed eosinophilic infiltration in all infants. Eosinophil counts range from 5 up to 45 per high powered field.

On EGD, 24/65 (36.92%) patients had evidence of hiatal hernia, 38/65 (58.46%) patients were normal, and 3/65 (4.61%) patients had esophageal erythema. Biopsies on EGD revealed eosinophilic infiltration in 49/65 (75.38%) patients. Eosinophil counts range from 9 up to 40 per high powered field.

Of the patients who underwent an esophageal pH study (total of 57/65), 32% were positive for gastroesophageal reflux.

Mesalamine treatment was given to 44 of the 65 patients. The mean age at which mesalamine was started was 3.5 months of age (range of 22 days old–7.5 months of age), and patients received mesalamine in a 40–60 mg/kg/day dose for an average of 100 ± 50 days.

Two patients required a second course of mesalamine. Two patients discontinued mesalamine due to one who developed a black tongue and one who had coughing that started on mesalamine and resolved on discontinuation.

All patients had a fecal occult blood test done before mesalamine was started. A positive fecal occult blood test (FOBT) was found in 35 (79.54%) of the 44 patients. On 15 patients, their FOBT changed from positive to negative after treatment with mesalamine. FOBT remained positive on two patients after treatment with mesalamine, and 11 patients who had a positive FOBT were not tested again after treatment.

Table 2 shows the percentage of patients that showed improvement or resolution of symptoms after 3 months from the initial evaluation. Patients who received treatment with mesalamine showed significant statistical improvement in fussiness/irritability symptoms, spitting up/vomiting, changes in stool consistency, decreased appetite/refusal to eat, respiratory problems, choking/gagging, back arching, and gassiness compared with patients without mesalamine. There was no statistical significance for symptoms of skin problems, blood or mucous in stool, hiccups, and sleep problems.

**TABLE 2.** Percentage of patients that showed improvement or resolution of symptoms after 3 months from the initial evaluation

Symptoms	With mesalamine treatment, No. of patients (%)	Without mesalamine treatment, No. of patients (%)	Statistical significance, *P*
Fussiness	30/41 (73.17%)	2/19 (10.52%)	0.0001
Spitting up/vomiting	32/44 (72.72%)	2/21 (9.5%)	0.0001
Changes on stool consistency*	23/44 (52.27%)	1/18 (5.2%)	0.0005
Decreased appetite/refusal to eat	19/24 (79.16%)	1/8 (12.5%)	0.0016
Respiratory problems†	23/26 (88.46%)	4/10 (40%)	0.0062
Choking/gagging	31/38 (81.57%)	5/13 (38.46%)	0.0106
Gassiness	15/29 (51.72%)	0/8 (0%)	0.0121
Back arching	22/23 (95.65%)	6/10 (60%)	0.0214
Skin problems‡	10/44 (58.82%)	4/8 (50%)	1.0000
Hiccups	30/31 (96.77%)	9/11 (81.81%)	0.1629
Sleeping difficulties	11/13 (84.61%)	0/2 (0%)	0.0571
Visible blood or mucous in stool	30/37 (81%)	5/7 (71.42%)	0.6188

*Watery or hard stools.

†Symptoms of persistent cough and congestion not related to another pathology.

‡Symptoms of atopic dermatitis and xerosis cutis.

In addition, the mesalamine group was less likely to be diagnosed with failure to thrive (20.45% vs. 47.61%) and less likely to report problems after the introduction of cow’s milk (85% vs. 22%) or soy milk (42.3% vs. 22 %) at 15 and 14 months, respectively.

Before mesalamine, 84% of patients were also receiving treatment with a histamine H2 antagonist (HH2A) and 53% with a proton pump inhibitor (PPI). After initiation of mesalamine treatment, HH2A treatment decreased to 57% and PPI treatment to 25%.

## DISCUSSION

While the severe cases we describe in this study share characteristics with patients diagnosed with food protein-induced enterocolitis syndrome (FPIES), they did not meet all of the criteria. FPIES is another non-IgE-mediated food-allergic severe enterocolitis. In contrast with FPIAP, these patients have an acute severe reaction to the allergen’s exposure, and they often require hospitalization due to the extreme severity of their symptoms. Infants with FPIES are described as dusky, pale, and limpness. They have profuse repetitive emesis, severe diarrhea, bloody stools, abdominal distention, lethargy, failure to thrive, severe dehydration to hypovolemic shock, hypotension, temperature instability, metabolic acidosis, anemia, eosinophilia, hypoalbuminemia, and methemoglobinemia (4).

Most of the patients referred to our clinic for FPIAP showed improvement or resolution of symptoms after protein elimination from maternal diet or change to a hypoallergenic or elemental formula. Our study focused on patients with persistent severe symptoms despite using an elemental formula for at least 2 weeks. We concluded that the addition of a short course of mesalamine was safe and effective in improving the most common symptoms found in our cohort. From the group of infants that received mesalamine, 80% had a positive fecal occult blood test before treatment, with most of them showing resolution after completing treatment.

An incidental finding was that despite nearly 100% clinical reflux symptoms in all the severe cases, only 32% had a positive pH impedance study. The improvement of symptoms related to gastroesophageal reflux disease after the treatment with mesalamine resulted in a decreased use of HH2A and PPI medications.

Compared to the most common unflavored milk alternatives such as almond milk, whole milk, and soy milk contain more protein per cup, making them ideal for fast-growing toddlers (8). It was interesting to find that patients who received mesalamine treatment were less likely to report any problem after transitioning to whole milk or soy milk after 1 year.

The FOBT is commonly used to diagnose FPIAP, and to monitor for treatment effectiveness. Our study showed that 78% of our severe cases had a positive FOBT despite all of them having evidence of proctocolitis in flexible sigmoidoscopy. Recent studies have similar results (9), leading to the conclusion that FOBT should not be used as the sole criteria to monitor treatment effectiveness in FPIAP.

Studies distinguishing normal from abnormal numbers of colonic and duodenal eosinophilia are scarce, and they often show variable results in healthy children versus in association with pathologies (10). Little is known about the prognosis or pathogenesis of the number of eosinophils found in biopsies for patients diagnosed with FPIAP. Our study found that most patients with severe refractory symptoms of FPIAP had an average of 20 eosinophils/hpf in both the duodenum and rectosigmoid colon.

More studies need to be done to understand the pathophysiology, diagnosis, and management of FPIAP and discover new potential treatments that would benefit the severe cases that fail to improve with standard therapies.

## References

[1] Arik Yilmaz E Soyer O Cavkaytar O. Characteristics of children with food protein-induced enterocolitis and allergic proctocolitis [published correction appears in Allergy Asthma Proc. 2017 Mar 1;38(2):19] Allergy Asthma Proc. 2017; 38:54–62 10.2500/aap.2017.38.4023 28052802

[2] Kaya A Toyran M Civelek E. Characteristics and prognosis of allergic proctocolitis in infants. J Pediatr Gastroenterol Nutr. 2015; 61:69–73 26039942 10.1097/MPG.0000000000000767

[3] Tsabouri S Nicolaou N Douros K. Food protein induced proctocolitis: a benign condition with an obscure immunologic mechanism. Endocr Metab Immune Disord Drug Targets. 2017; 17:32–37 28376708 10.2174/1871530316666170331165356

[4] Nowak-Węgrzyn A. Food protein-induced enterocolitis syndrome and allergic proctocolitis. Allergy Asthma Proc. 2015; 36:172–184 25976434 10.2500/aap.2015.36.3811 PMC4405595

[5] Lake AM. Food-induced eosinophilic proctocolitis. J Pediatr Gastroenterol Nutr. 2000; 30 (Suppl S58–S60 10634300 10.1097/00005176-200001001-00009

[6] Winter HS Antonioli DA Fukagawa N. Allergy-related proctocolitis in infants: diagnostic usefulness of rectal biopsy. Mod Pathol. 1990; 3:5–10 2308921

[7] Goldman H Proujansky R. Allergic proctitis and gastroenteritis in children. Clinical and mucosal biopsy features in 53 cases. Am J Surg Pathol. 1986; 10:75–86 3953938 10.1097/00000478-198602000-00001

[8] Porto A Drake R. Parent FAQs: Cow’s Milk Alternatives. SearchEngineWatch Web site: https://www.healthychildren.org/English/healthy-living/nutrition/Pages/milk-allergy-foods-and-ingredients-to-avoid.aspx. Published October 13, 2017. Accessed July 20, 2020

[9] Concha S Cabalín C Iturriaga C. [Diagnostic validity of fecal occult blood test in infants with food protein-induced allergic proctocolitis]. Rev Chil Pediatr. 2018; 89:630–637 30571806 10.4067/S0370-41062018005000901

[10] Mark J Fernando SD Masterson JC. Clinical Implications of Pediatric Colonic Eosinophilia. J Pediatr Gastroenterol Nutr. 2018; 66:760–766 29095349 10.1097/MPG.0000000000001784 PMC5916023

